# A customised 3D U-Net model for bifid mandibular canal segmentation and detection on CBCT: a diagnostic accuracy study

**DOI:** 10.1186/s12903-026-08336-0

**Published:** 2026-04-23

**Authors:** Sara Aboseif, Enas Anter, Esraa Khairy

**Affiliations:** Department of Oral and Maxillofacial Radiology, Faculty of Dentistry, Cairo University, Cairo, Egypt

**Keywords:** Deep learning, Artificial intelligence, Cone-beam computed tomography, Inferior alveolar nerve, Bifid mandibular canal, Mandibular canal, 3D U-Net

## Abstract

**Background:**

Bifid mandibular canals (BMCs) represent a clinically significant anatomical variation that increases the risk of iatrogenic inferior alveolar nerve injury during oral and maxillofacial procedures. Accurate identification of BMCs on cone-beam computed tomography (CBCT) remains challenging due to their subtle morphology and reliance on observer expertise. This study aimed to evaluate the diagnostic performance of a customised deep learning–based three-dimensional (3D) U-Net model for the automated delineation and detection of BMCs on CBCT volumes.

**Methods:**

A total of 208 CBCT volumes acquired using the Planmeca ProMax 3D MID system (Planmeca Oy, Helsinki, Finland) were retrospectively selected. The dataset was stratified into a training set, intentionally enriched with anatomical variants, and an independent test set. Ground truth annotations were established through manual voxel-wise segmentation by experienced oral and maxillofacial radiologists. A customised 3D U-Net architecture was utilised via the MONAI framework with five-fold cross-validation. Model performance was evaluated on the independent test set using voxel-wise metrics, including the Dice Similarity Coefficient (DSC) and Intersection-over-Union (IoU), as well as case-level detection metrics (sensitivity and specificity).

**Results:**

The model achieved mean DSC values of 0.91 for standard mandibular canals and 0.77 for bifid variants. On the independent test set, enriched with complex morphological subtypes, the model demonstrated 100% sensitivity, 81.8% specificity, and 90.5% overall diagnostic accuracy for detecting BMCs.

**Conclusions:**

This study demonstrates that a customised 3D U-Net framework can accurately identify BMCs on CBCT volumes within a single-centre setting. When used as a decision-support tool, the proposed approach has the potential to assist radiologists in recognising anatomically complex canal variants. Further multicentre validation is required to confirm broader clinical applicability.

## Background

The integration of artificial intelligence (AI) into dentistry has significantly improved diagnostic accuracy and treatment planning across multiple dental specialities, including oral and maxillofacial radiology [1]. The bifid mandibular canal (BMC) is an anatomical variation that presents a significant risk to the success of various dental procedures, including wisdom tooth extraction, implant placement [2], nerve block anaesthesia [3] and osteotomy [4]. Its diverse morphology complicates recognition on imaging. Numerous classification systems have been proposed to account for these variations, ranging from simple bifurcations to complex multi-branched networks, further emphasising the diagnostic challenge it poses [5].

Since its first description in 1973 [6], the reported prevalence of BMC has varied significantly based on the imaging modality used for diagnosis. Conventional panoramic radiographs detected bifid mandibular canal (BMCs) in 0.35% to 2% of cases [7–9], while digital panoramic imaging showed a slightly higher rate of 2.3% [10]. However, with the introduction of cone-beam computed tomography (CBCT), prevalence estimates have increased dramatically to over 60% in some studies [11, 12]. This rise is attributed to CBCT’s superior diagnostic capabilities compared to panoramic radiography, as it offers three-dimensional visualisation [13], provides isotropic sub-millimetre resolution, and eliminates structural superimposition, while also delivering a substantially lower radiation dose than conventional computed tomography (CT) [14]. Despite CBCT’s advanced features, such as multi-planar reconstruction (MPR) and semi-automated nerve tracing tools, accurate identification of the BMC remains challenging due to its intricate course, making detection strongly dependent on the clinician’s expertise [15].

The accurate detection of the mandibular canal (MC) on CBCT largely depends on the clarity of its cortical boundaries and the surrounding bone structure. Consequently, MC identification remains challenging in nearly 20% of patients [16]. Factors such as variations in CBCT acquisition protocols [17], inherent artefacts, image noise, and the absence of standardised Hounsfield units may further compromise the visibility and precise annotation of the canal [18]. These limitations have driven the development of deep learning (DL) models capable of automating segmentation and detecting anatomical variations within volumetric datasets of significant clinical importance [19].

Several DL models, such as U-Net, 3D U-Net, V-Net, Attention U-Net, and DenseNet-based architectures, have been successfully applied to the automatic segmentation of the MC on CBCT scans. These models have demonstrated high accuracy and robustness in detecting complex canal morphologies, including bifid configurations, and have contributed significantly to enhancing diagnostic reliability in maxillofacial imaging [15]. They offer a faster, more consistent alternative to manual tracing, minimising observer fatigue and variability [20]. However, the performance of such systems may be influenced by population-specific anatomical variation, emphasising the need for diverse training datasets [21].

Although the role of AI in general MC detection has been explored, research specifically targeting BMCs remains limited [15]. Therefore, this study was designed to address this gap in knowledge by evaluating the diagnostic accuracy of a customised DL-based segmentation model for detecting BMCs on CBCT volumes. To evaluate its clinical potential, the model outputs were assessed against expert-generated segmentations, which served as the reference standard.

## Methods

### Study design and dataset collection

This retrospective study was approved by the Research Ethics Committee, Faculty of Dentistry, Cairo University (approval No. 32525). Owing to the retrospective design and anonymisation of all scans, the requirement for individual informed consent was waived. A total of 208 anonymised CBCT volumes were retrieved from the institutional digital archive of the Faculty of Dentistry, Cairo University, encompassing scans acquired between February 2023 and February 2025.

All scans were acquired at a single institution using the same CBCT system (Planmeca ProMax 3D MID, Planmeca Oy, Helsinki, Finland). As this was a retrospective study, acquisition parameters were not standardised but reflected routine clinical practice at the time of imaging. Tube voltage and tube current were 90 kVp and 8 mA, respectively, while exposure time varied automatically (12–13 s) according to the selected field of view. Voxel size ranged between 0.3 and 0.4 mm, and the field of view (FOV) varied between scans depending on the clinical indication.

Patients aged 15 years or older were eligible, provided that scans demonstrated the full course of at least one mandibular canal, from the mandibular foramen to the mental foramen. Exclusion criteria comprised severe artefacts interfering with canal tracing, incomplete canal coverage, or significant mandibular deformities.

### Sample size estimation

The sample size was estimated using previously reported mandibular canal segmentation performance as a surrogate benchmark, due to the absence of prior deep learning studies specifically addressing bifid mandibular canal detection. Based on a reported Dice Similarity Coefficient of 0.9248 (SD = 0.033) [22], a 95% confidence level, and a margin of error of 0.006, the estimated minimum sample size was 117 CBCT volumes. To enhance model robustness and internal validity, a larger dataset of 208 CBCT scans was ultimately used. The sample size estimation was reviewed and approved by the Medical Biostatistics Unit, Faculty of Dentistry, Cairo University.

### Dataset composition and morphological distribution

The total dataset (*n* = 208) was divided into two distinct subsets: a training/validation set (*n* = 187), on which five-fold cross-validation was exclusively performed, and a strictly held-out test set (*n* = 21) reserved for independent evaluation.

To ensure the model learned to handle complex anatomical variations, the training set was intentionally enriched with variant cases rather than reflecting a natural population distribution. Consequently, BMCs were present in 145 training cases (77.5%), while the remaining 42 scans (22.5%) exhibited standard single canals.

Within the training set, a total of 171 BMC sides were identified and classified according to Naitoh et al. [11]. The distribution was as follows:

- Retromolar: 58 sides (33.9%) (Fig. 1a)
  **Fig. 1:**
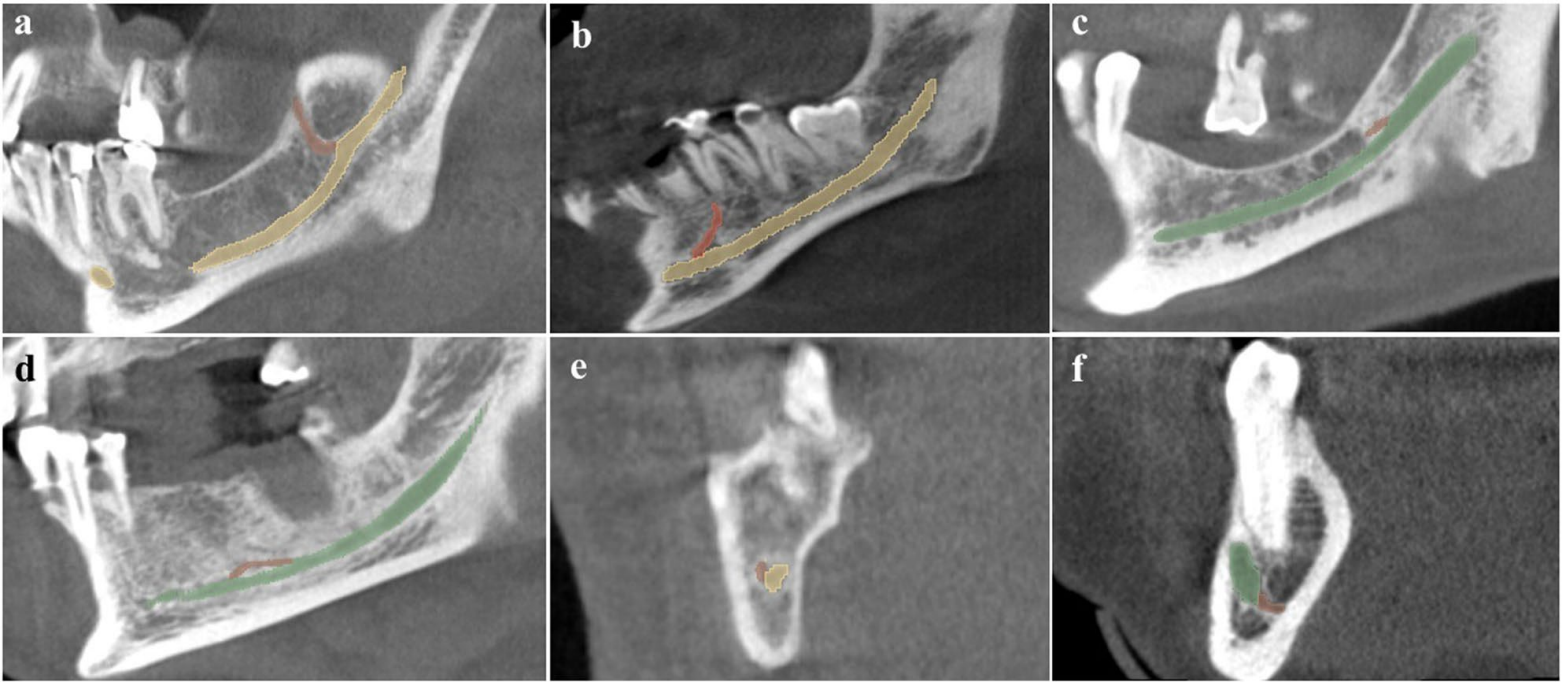
3D segmentation of bifid mandibular canal types in 3D Slicer software (Yellow/Green: main canal; Red: bifid variant). **a** Retromolar; (**b**) Dental; (**c**) Forward without confluence; (**d**) Forward with confluence; (**e**) Buccal; (**f**) Lingual
- Dental: 53 sides (31.0%) (Fig. 1b)
- Forward: 45 sides (26.3%) — comprising 33 sides without confluence (Fig. 1c) and 12 with confluence (Fig. 1d)
- Buccolingual: 15 sides (8.8%) — comprising 4 buccal (Fig. 1e) and 11 lingual canals (Fig. 1f)

This enriched distribution was implemented solely for model training purposes and does not reflect the true population prevalence of BMCs. No cases meeting the criteria for a true trifid mandibular canal (TMC) configuration were identified in the present dataset. Mandibular canal variants were therefore classified using this CBCT-based framework, which encompasses bifid but not trifid morphologies.

To rigorously evaluate the model’s performance across diverse anatomical configurations, the independent test set (*n* = 21) was stratified to represent specific anatomical challenges rather than being randomly sampled. The 10 positive cases in the test set were selected to represent the full spectrum of BMC morphologies, including 3 dental, 2 retromolar, 4 forward variants (two with confluence), and 1 buccolingual subtype. This design enabled targeted assessment of model behaviour across distinct and anatomically challenging variants, rather than predominantly common presentations.

### Ground truth annotation

Ground truth labels were generated through manual voxel-wise segmentation using 3D Slicer (version 5.8; The Slicer Community, Boston, MA, USA). Two oral and maxillofacial radiologists with different levels of clinical experience (3 and 11 years, respectively) independently traced the right and left mandibular canals in axial, sagittal, and coronal planes. Each CBCT volume was additionally labelled at the patient level as “Present” or “Absent” with respect to the presence of BMCs.

In cases of annotation disagreement, discrepancies were resolved through discussion between the two annotators to establish a single reference standard for model training and evaluation. As the final ground truth was derived from this consensus process rather than parallel independent labels, formal inter-rater agreement statistics were not calculated. This dual-review approach minimised observer bias and ensured anatomically consistent reference segmentations.

### Model architecture and implementation

A 3D U-Net was developed from scratch using PyTorch and the MONAI framework. The model incorporated residual encoder blocks to improve feature propagation and gradient stability. The model outputs multiclass segmentation masks distinguishing between four classes: background, right MC, left MC, and bifid branches. This multiclass formulation preserves canal laterality and enables simultaneous segmentation of standard and bifid canals within a single output, supporting anatomically detailed assessment beyond what a binary detection approach can provide.

**Fig. 2:**
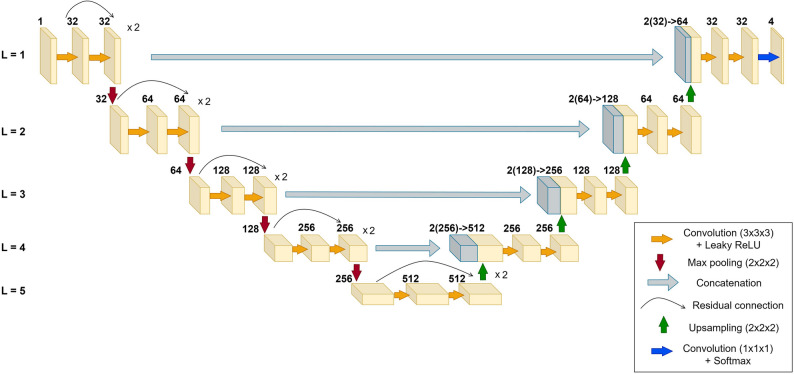
Schematic architecture of the proposed custom 3D U-Net. The network features a 5-level residual encoder-decoder structure designed for multiclass segmentation. Key components include residual connections for feature extraction and skip connections for spatial recovery, culminating in a softmax output layer. The encoding blocks in levels 1 through 5 are stacked with a multiplier of 2 (×2) to achieve a total depth of 26 convolutional layers

Inputs were normalised using Z-score normalisation. Leaky ReLU was used for hidden activations, and a softmax layer was applied at the output. A customised 3D U-Net architecture was implemented, consisting of five encoder blocks and five decoder blocks. Each encoder block contained multiple residual convolutional layers, resulting in a total of 26 convolutional layers in the encoding pathway. This deeper design enables the model to capture subtle anatomical features such as BMCs. The detailed architecture of the 3D U-Net is illustrated (Fig. 2).

### Training strategy

Training was performed using randomly sampled patches (64 × 128 × 128 voxels) centred on manually annotated canal labels to ensure anatomical relevance. This strategy was adopted because the mandibular canal is a highly sparse structure within CBCT volumes; purely random patch sampling would therefore result in extreme class imbalance, with a predominance of background-only patches and insufficient representation of bifid branches.

Although patches were centred on canal labels, each patch remained dominated by non-target voxels, including trabecular bone, marrow spaces, and air, thereby preserving substantial background context. No fixed patch-level class ratio was imposed; instead, patch composition varied dynamically across epochs and cross-validation folds according to case-level sampling. Patches were not tightly cropped to the canal lumen, and no background-only patches were explicitly excluded during training. Patch sampling therefore reflected the case-level composition of the intentionally enriched training dataset, with proportional representation of bifid and standard canal cases during training rather than population prevalence.

**Fig. 3:**
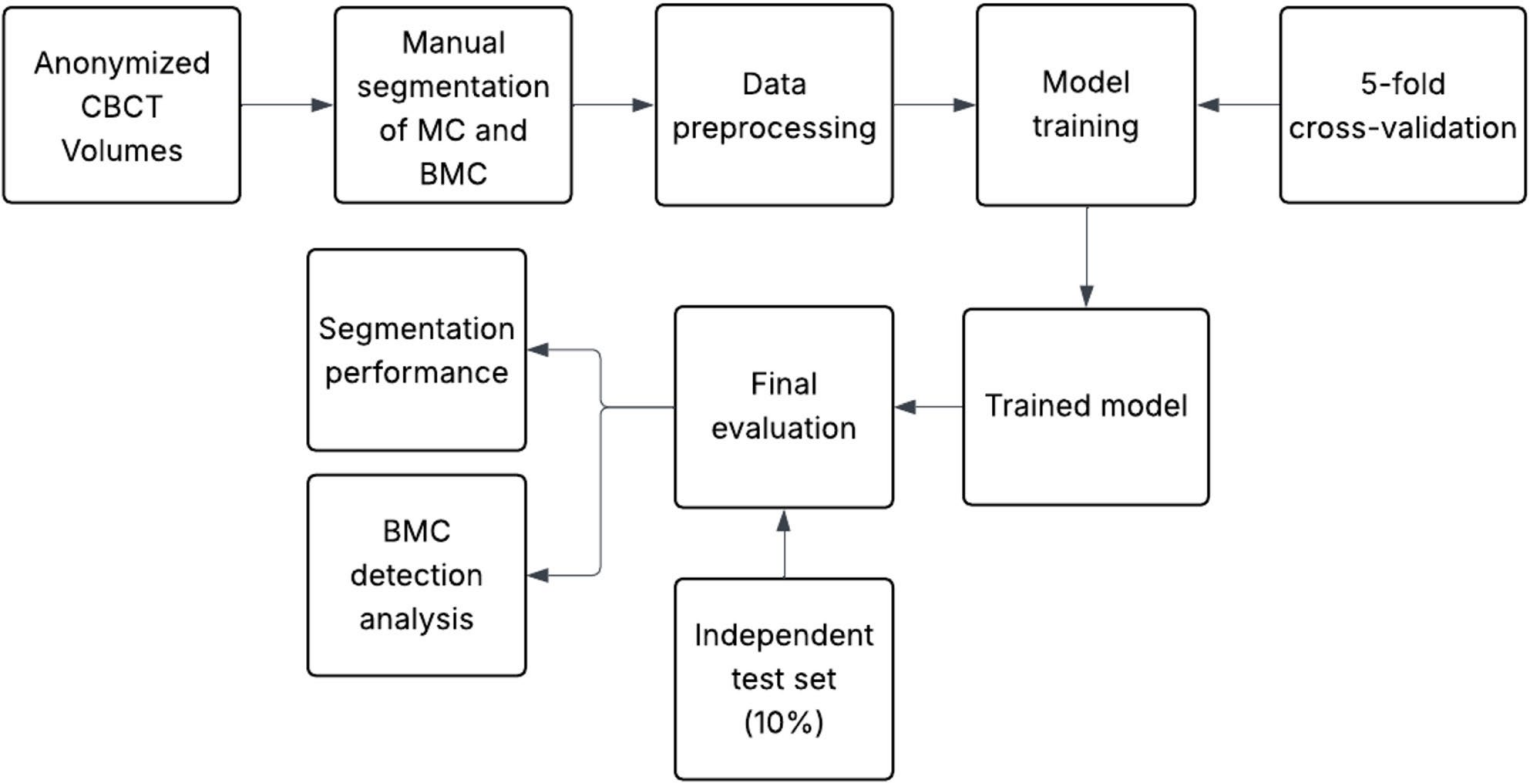
Workflow of the study. The pipeline illustrates the progression from data acquisition and manual segmentation to model training, cross-validation, and final independent evaluation

The network was trained for 1000 epochs with a batch size of 2 using the AdamW optimiser and a linear learning rate decay schedule. Cross-entropy loss was employed for optimisation. To mitigate overfitting, dropout (10%) and five-fold cross-validation were applied, ensuring balanced representation of bifid and non-bifid cases across folds. Five-fold cross-validation was performed exclusively on the training set for model selection and performance estimation, and random seeds were fixed to ensure reproducibility. An overview of the workflow is provided in Fig. 3.

**Fig. 4:**
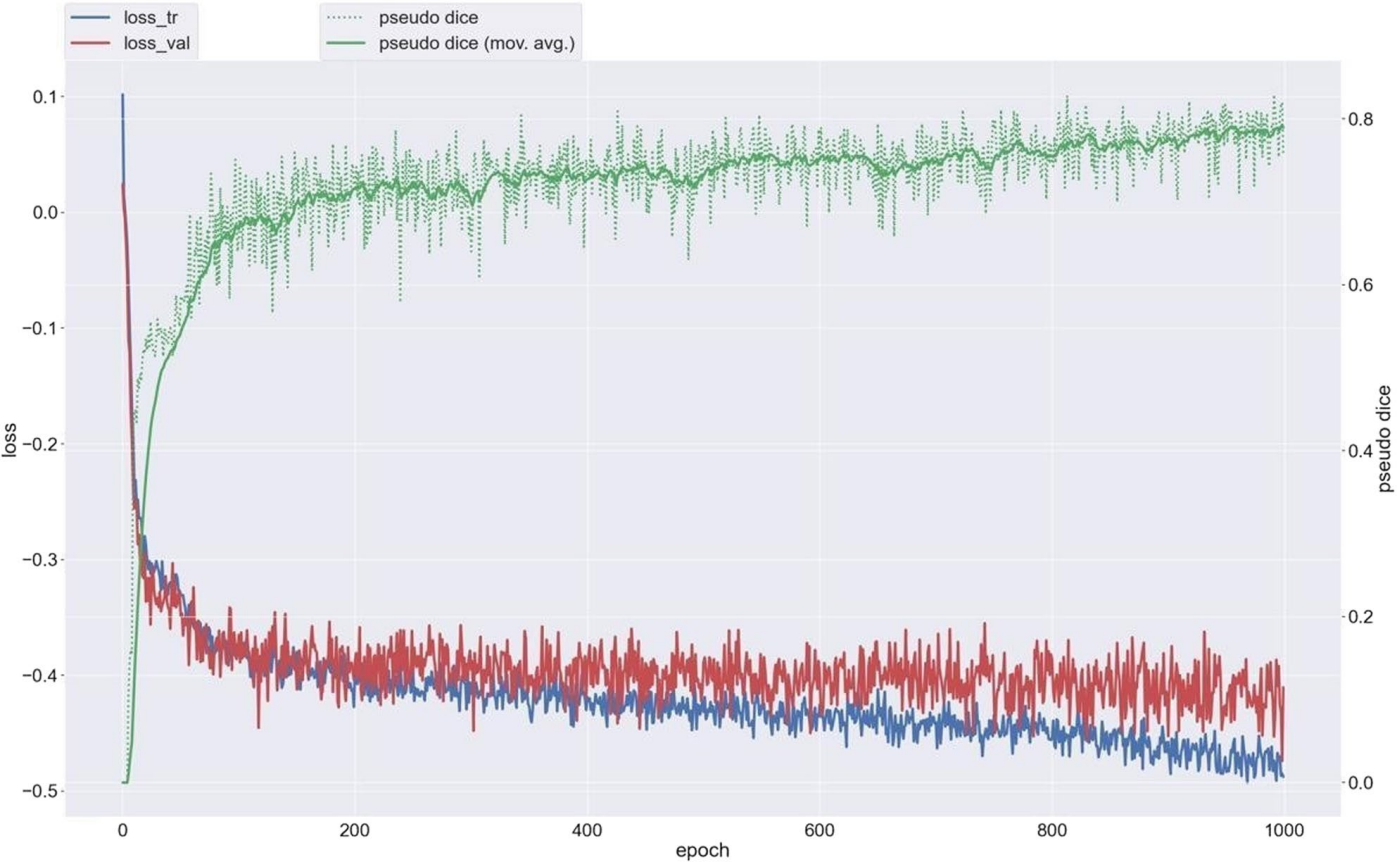
Training and validation loss curves and pseudo-Dice scores across training epochs, demonstrating stable convergence without late-epoch divergence over the full training schedule

The final model was selected based on the epoch achieving the highest mean validation Dice score across cross-validation folds. Early stopping was not applied; instead, model selection was performed retrospectively based on validation performance. Training stability was assessed by monitoring training and validation loss curves and pseudo-Dice scores across epochs (Fig. 4). Both losses demonstrated comparable convergence behaviour without late-epoch divergence, indicating stable optimisation across the training schedule.

### Data augmentation and post-processing

**Fig. 5:**
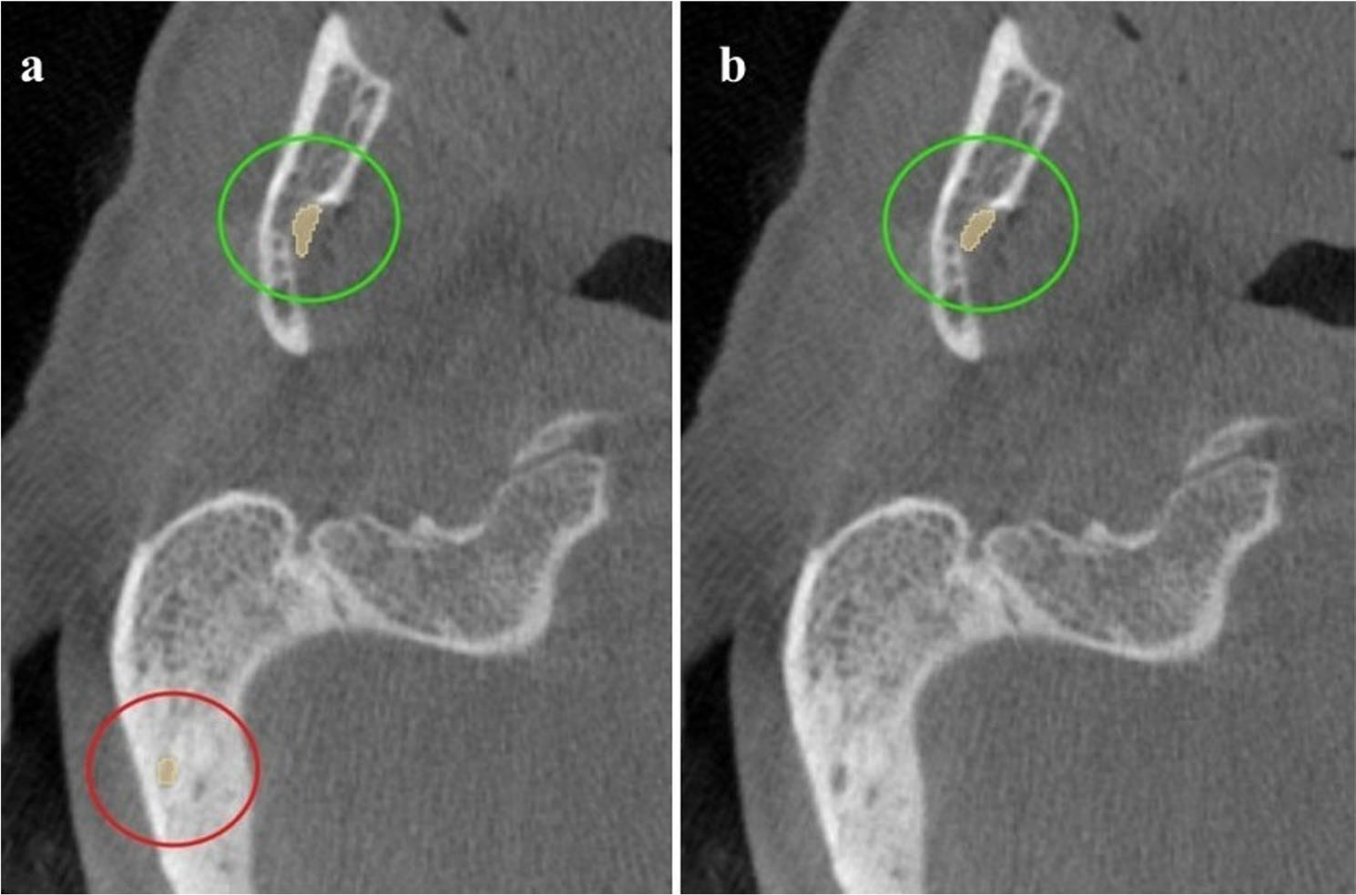
Axial CBCT slices showing post-processing effects on segmentation: (**a**) initial prediction with an extraneous component (red circle); (**b**) final output after removal, retaining only the mandibular canal (green circle)

During training, axial flipping and rotations were applied as geometric augmentations to improve model robustness to spatial variability and reduce overfitting. Elastic deformation was avoided to preserve the true anatomical linearity of the canal structures, and no intensity shifts or affine scaling were employed. For inference, a sliding window strategy was implemented with a 30% overlap between adjacent patches to maintain spatial continuity across boundaries. All inference was performed fully on GPU using an NVIDIA RTX 3090 (24 GB VRAM), without CPU offloading or gradient computation. Memory usage remained within available GPU resources during sliding-window reconstruction, and no model sharding or patch aggregation outside GPU memory was required. Following inference, post-processing was performed using connected-component analysis to remove spatially isolated components that did not share a point of connectivity with the predicted mandibular canal and were therefore anatomically implausible. Because true bifid branches originate from the main mandibular canal before diverging, only components without any connection to the main canal were removed, as illustrated in Fig. 5.

### Statistical analysis

Statistical evaluation was conducted using Python (v3.9) with the SimpleITK and NumPy libraries. Prior to analysis, all predictions were resampled to match reference voxel sizes, rigidly registered, and label indices harmonised. This study was designed as a diagnostic accuracy evaluation rather than a comparative or superiority study; therefore, formal hypothesis testing between models or against human readers was not performed.

### Voxel-wise segmentation metrics

The following metrics were computed for right MC, left MC, and BMCs:

- Dice Similarity Coefficient (DSC) = $\:\frac{(2\:\times\:\:\mathrm{T}\mathrm{P})}{(2\:\times\:\:\mathrm{T}\mathrm{P}\:+\:\mathrm{F}\mathrm{P}\:+\:\mathrm{F}\mathrm{N})}$
- Intersection over Union (IoU) = $\:\frac{\mathrm{T}\mathrm{P}}{(\mathrm{T}\mathrm{P}\:+\:\mathrm{F}\mathrm{P}\:+\:\mathrm{F}\mathrm{N})}$
- Precision = $$\:\frac{\mathrm{T}\mathrm{P}}{(\mathrm{T}\mathrm{P}\:+\:\mathrm{F}\mathrm{P})}$$
- Recall (Sensitivity) = $$\:\frac{\mathrm{T}\mathrm{P}}{(\mathrm{T}\mathrm{P}\:+\:\mathrm{F}\mathrm{N})}$$

where TP = true positives, FP = false positives, FN = false negatives. Scans with no positive voxels in both prediction and reference were excluded for each class.

### Case-level detection metrics

Binary classification was performed at the whole-volume level (per patient) to determine the presence of a bifid mandibular canal. A CBCT volume was classified as ‘positive’ if the total number of voxels predicted for the bifid class exceeded 20 voxels, regardless of laterality (unilateral or bilateral). This volumetric threshold was derived from a retrospective analysis of manually segmented bifid mandibular canals, in which the smallest confirmed anatomical branch comprised 42 voxels. A conservative cut-off of 20 voxels was therefore selected to suppress isolated noise artefacts while preserving small, valid canal segments. Metrics included:

- Sensitivity = $$\:\frac{\mathrm{T}\mathrm{P}}{\left(\mathrm{T}\mathrm{P}+\mathrm{F}\mathrm{N}\right)}$$
- Specificity = $$\:\frac{\mathrm{T}\mathrm{N}}{\left(\mathrm{T}\mathrm{N}+\mathrm{F}\mathrm{P}\right)}$$
- Accuracy = $$\:\frac{(\mathrm{T}\mathrm{P}+\mathrm{T}\mathrm{N})}{\left(\mathrm{T}\mathrm{P}+\:\mathrm{T}\mathrm{N}+\mathrm{F}\mathrm{P}+\mathrm{F}\mathrm{N}\right)}$$
- Positive Predictive Value (PPV) = $\:\frac{\mathrm{T}\mathrm{P}}{\left(\mathrm{T}\mathrm{P}+\:\mathrm{F}\mathrm{P}\right)}$
- Negative Predictive Value (NPV) = $\:\frac{\mathrm{T}\mathrm{N}}{\left(\mathrm{T}\mathrm{N}+\mathrm{F}\mathrm{N}\right)}$
- Area Under the Curve (AUC): derived from the ROC curve, plotting sensitivity against 1–specificity.

### Surface deviation analysis

Qualitative deviations were visualised using CloudCompare (v2.13.2), with colour-coded Euclidean distances demonstrating over-segmentation, under-segmentation, and optimal overlap. Optimal overlap was defined as a surface deviation of < 1 mm (green). Under-segmentation (blue) and over-segmentation (red) correspond to negative and positive deviations exceeding 1 mm, respectively.

## Results

### Voxel-wise segmentation performance

The model achieved high performance for right and left mandibular canal (MCs) (DSC ≈ 0.91, IoU ≈ 0.83) and clinically acceptable results for BMCs (DSC ≈ 0.77, IoU ≈ 0.64, recall ≈ 0.70). Full metrics are presented in Table 1.

**Table 1:** Segmentation accuracy metrics per anatomical segment

Segment	Mean ± SD (95% CI)			
	Dice	IoU	Precision	Recall
Right MC	0.9067 ± 0.0303 (0.8921–0.9213)	0.8306 ± 0.0498 (0.8066–0.8546)	0.9288 ± 0.0504 (0.9046–0.9531)	0.8879 ± 0.0416 (0.8679–0.9080)
Left MC	0.9059 ± 0.0365 (0.8884–0.9235)	0.8300 ± 0.0602 (0.8009–0.8590)	0.9064 ± 0.0670 (0.8741–0.9387)	0.9083 ± 0.0253 (0.8961–0.9205)
BMC	0.7742 ± 0.0887 (0.7060–0.8424)	0.6389 ± 0.1151 (0.5505–0.7274)	0.8829 ± 0.0703 (0.8288 − 0.9369)	0.7008 ± 0.1328 (0.5987–0.8029)

*MC* Mandibular canal, *BMC* Bifid mandibular canal, *SD* Standard deviation, *CI* Confidence interval

**Fig. 6:**
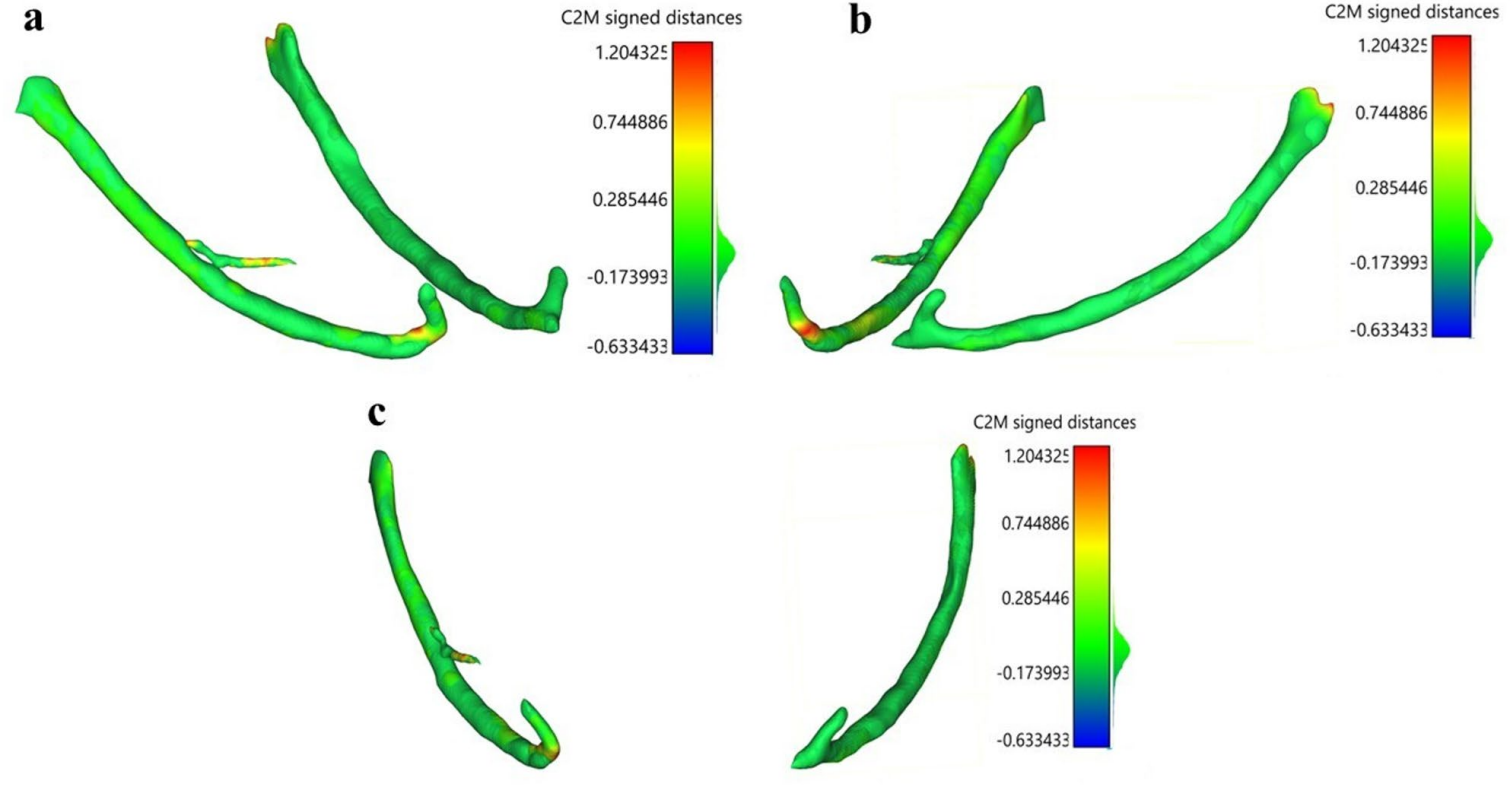
3D deviation maps comparing AI-segmented and expert-traced mandibular canals. Images are presented in right oblique (**a**), left oblique (**b**), and frontal (**c**) views. Color-coded Euclidean distances show over-segmentation (red), under-segmentation (blue), and optimal overlap (green)

Qualitative segmentation examples are illustrated, and deviation maps highlight discrepancies mainly near the anterior loop and bifurcation; most remained within submillimetre tolerance (Fig. 6).

### Case-level detection performance

On the held-out independent test set (*n* = 21), comprising 10 positive and 11 negative cases, the model correctly identified all BMC cases, yielding 10 true positives (TP), 9 true negatives (TN), 2 false positives (FP), and no false negatives (FN). This corresponded to a case-level sensitivity of 100.0% (95% CI: 72.2–100.0) and an overall diagnostic accuracy of 90.5% (95% CI: 71.1–97.3). Additional performance metrics, including positive predictive value (PPV) and negative predictive value (NPV), are summarised in Table 2.

**Table 2:** Diagnostic performance metrics for BMC detection

Metric	Value (%)	95% Confidence Interval
Sensitivity	100.0	[72.2–100.0]
Specificity	81.8	[52.3–94.9]
Positive Predictive Value (PPV)	83.3	[55.2–95.3]
Negative Predictive Value (NPV)	100.0	[70.1–100.0]
Overall Accuracy	90.5	[71.1–97.3]

Confidence intervals were calculated using the Wilson score method

### ROC analysis

**Fig. 7:**
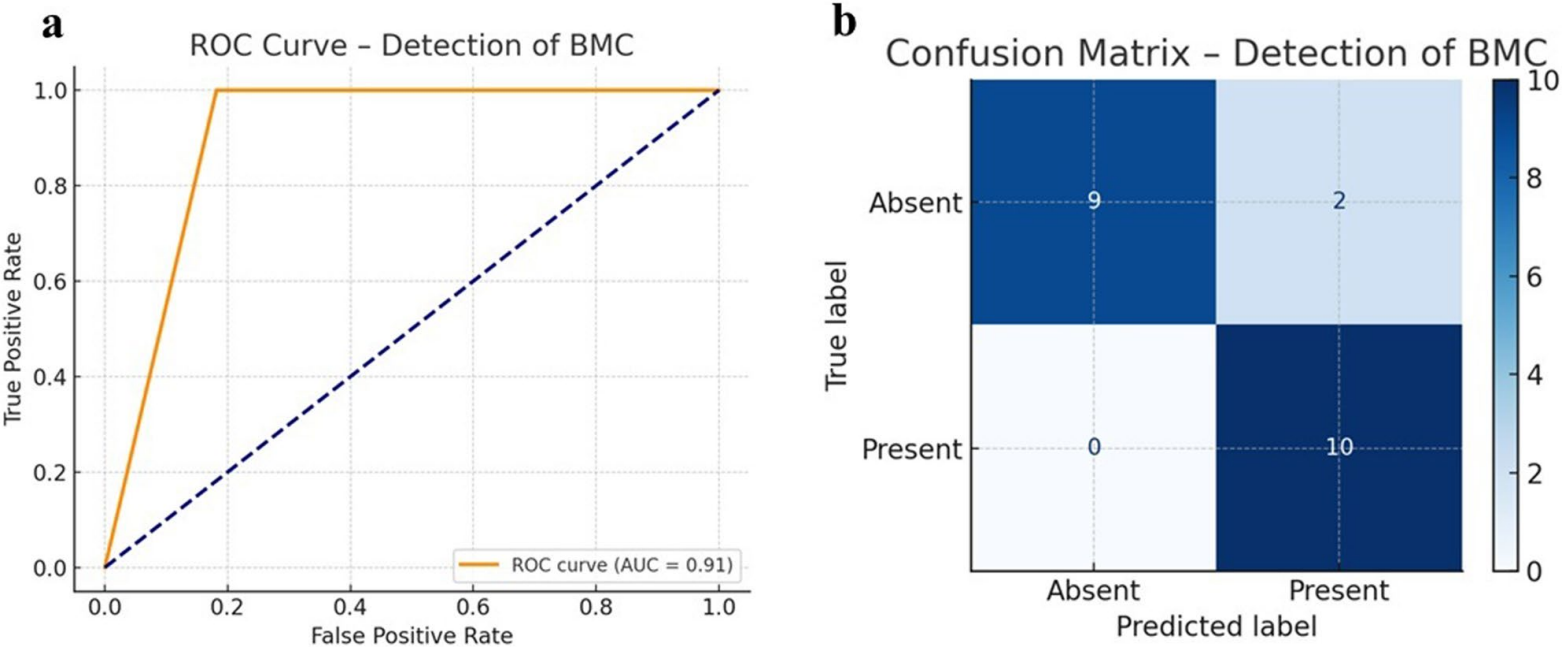
Diagnostic performance for bifid mandibular canal detection: (**a**) ROC curve with area under the curve (AUC) = 0.91; (**b**) confusion matrix (19/21 correct; 2 FP, 0 FN)

The ROC curve showed an AUC of 0.91 for BMC detection (Fig. 7a). Classification results are summarised in the corresponding confusion matrix (Fig. 7b).

## Discussion

Reliable assessment of the mandibular canal and its anatomical variations is essential for safe oral and maxillofacial surgical practice. Among these variations, bifid mandibular canals (BMCs) pose a particular diagnostic challenge, as their subtle branching patterns are frequently difficult to visualise on radiographs, even when advanced reconstruction tools are employed [15]. When unrecognised, these variants have been associated with complications including haemorrhage, postoperative paraesthesia, traumatic neuroma formation, and permanent neurosensory deficits [4, 23]. BMCs are also a recognised cause of failed inferior alveolar nerve block, particularly when conventional techniques such as the Halstead approach are used [24]. Early identification enables modification of anaesthetic strategies, including adoption of the Gow–Gates technique [25] or administration of injections at a lower level [8], thereby reducing the risk of intraoperative complications. Clinical case reports further emphasise this importance, documenting resolution of implant-related pain and successful exodontia following recognition of previously undetected BMCs [23, 26]. Collectively, these observations underscore the clinical need for an accurate and sensitive method for BMC detection.

Artificial intelligence offers a promising solution to this diagnostic challenge. Since its introduction, the U-Net architecture has been widely adopted in biomedical image analysis due to its ability to balance global contextual understanding with precise spatial localisation [19]. In the present study, a multiclass deep learning framework was applied to segment both standard MCs and BMCs on CBCT images. The model demonstrated strong voxel-wise performance for standard MCs (mean DSC ≈ 0.91, recall ≈ 0.90) and clinically acceptable performance for BMCs (DSC ≈ 0.77, recall ≈ 0.70). Sensitivity for BMC detection reached 100% within the independent test set, indicating that no bifid variants were missed in this cohort. This finding is clinically relevant in a preoperative diagnostic context, where false-negative results may carry significant clinical consequences.

Only a limited number of studies have explicitly addressed BMC segmentation, and these have relied on standardised deep learning frameworks. Gümüşsoy et al. [27] applied the self-configuring nnU-Net v2 to 69 CBCT volumes. While satisfactory performance was achieved for the standard MC (DSC ≈ 0.82), segmentation accuracy for bifid variants was substantially lower (DSC ≈ 0.46, recall ≈ 0.42). These findings highlight the ongoing challenge of accurately segmenting bifid mandibular canals, particularly when anatomical conspicuity is low, and underscore the influence of task formulation and dataset composition on reported performance. Similarly, Ye et al. [20] employed a conventional nnU-Net backbone on a larger dataset and reported a higher overall DSC (≈ 0.74); however, model performance was strongly dependent on canal conspicuity. Recall reached 91.3% for clearly visible bifurcations but declined to 58.1% for subtle or obscure variants. Importantly, neither study incorporated architectural adaptations explicitly targeted at enhancing detection of these faint structures.

A deep residual 3D U-Net was implemented in this study as a well-established backbone for volumetric medical image segmentation. The network comprised 26 convolutional layers, with residual connections incorporated to support stable optimisation of increased depth in accordance with the principles described by He et al. [28]. Prior studies addressing BMC segmentation have employed established architectures, including nnU-Net-based configurations [20, 27], highlighting the ongoing challenge of accurately delineating subtle bifid canal morphology on CBCT. Within the context of the present task and dataset, the residual 3D U-Net provided sufficient representational capacity to delineate fine anatomical features of BMCs, without introducing additional architectural complexity or asserting architectural superiority.

To fully exploit this architectural capacity, both the training and independent test datasets were purposively stratified to include rare and diagnostically challenging BMC subtypes, including buccolingual (≈ 0.8%) and dental (≈ 2.8%) canals. Forward canals, present in approximately 4.7% of hemi-mandibles and often underrepresented in prior datasets, were also incorporated to mitigate class imbalance [29]. The combination of targeted sampling and task-specific implementation enabled consistent evaluation of model behaviour across a range of diagnostically challenging bifid canal morphologies within the available dataset. From a clinical perspective, this approach may support greater confidence during surgical planning in regions where BMCs are most prevalent, particularly within the posterior mandible [30]. Trifid mandibular canals (TMCs) have been reported far less frequently than bifid variants in CBCT-based studies. Recent CBCT-based evidence indicates that TMCs are uncommon, with reported prevalence of approximately 1–2% in CBCT series, depending on imaging voxel size and acquisition parameters [31]. The absence of TMCs in the present dataset is therefore plausible and consistent with this low reported prevalence, placing trifid variants outside the scope of the current analysis.

Several studies have applied AI to MC segmentation without extending their scope to bifid variants. Semi-automated U-Net-based approaches have reported moderate performance (DSC ≈ 0.78) [32], while fully automated pipelines achieved higher accuracy for standard MCs (DSC ≈ 0.89) [33]. In comparison, the present model achieved comparable or superior performance for standard MCs (DSC ≈ 0.91, recall ≈ 0.89), likely reflecting differences in annotation strategy and the use of full-arch voxel-level training. More specialised frameworks, such as combinations of ResNet-50 with U-Net++, have demonstrated DSC values exceeding 0.92 for MC segmentation [22]. However, these approaches were restricted to the third molar region. By contrast, the present multiclass framework achieved competitive accuracy for standard MCs while uniquely incorporating BMCs across the entire mandibular arch, thereby extending applicability to a broader range of surgical contexts.

Annotation methodology represents another determinant of segmentation performance. Di Bartolomeo et al. [34] demonstrated that dense voxel-wise annotations (DSC ≈ 0.79) outperform sparse or circular approximations (DSC ≈ 0.52–0.69), while substantially reducing segmentation time. The findings of the present study support this observation, as voxel-wise labelling preserved canal morphology and angulation, enabling the network to capture bifid branches that might be overlooked by tracing-based or simplified annotation approaches. From a surgical perspective, such precision is critical, as millimetre-level discrepancies can significantly affect implant safety margins and increase the risk of nerve injury.

Beyond segmentation accuracy, computational efficiency is a key determinant of clinical feasibility. Manual inspection and dense tracing of the MC may require up to 50 min per case, whereas the proposed model generated complete voxel-wise segmentations within approximately 10–12 min. This represents a reduction of 38–40 min per scan (76–80%), constituting a substantial saving in radiologist time. Although this runtime exceeds that reported in some studies [32, 34], those shorter processing times were achieved through methodological simplifications, such as restricting the field of view or employing circular canal approximations. In contrast, the present approach performed full-volume voxel-wise segmentation, preserving subtle bifid branches and more closely reflecting real-world clinical evaluation. While computationally demanding, this strategy prioritises anatomical fidelity and yields a clinically meaningful reduction in interpretation time relative to manual methods.

To the authors’ knowledge, this study represents one of the first applications of a multiclass 3D convolutional framework for simultaneous voxel-wise segmentation of standard and BMCs across the full mandibular arch on CBCT. Unlike previous work relying on generic nnU-Net configurations or focusing exclusively on standard canals or third molar regions, the present framework integrates dense voxel-wise annotation, anatomically guided patch-based training, and class-specific post-processing. These design choices supported reliable detection of subtle bifid branches within the evaluated dataset and facilitated complete identification of bifid variants on the independent test set. By adopting a multiclass segmentation framework, this work enables joint representation of canal laterality and bifid branching patterns within a single inference pipeline, supporting anatomically detailed assessment.

Several limitations should be acknowledged. First, although annotations were independently performed and resolved through consensus, inter-observer agreement prior to consensus was not quantitatively assessed. This limits formal evaluation of annotation reliability. Second, all CBCT volumes were acquired using a single imaging system (Planmeca ProMax 3D MID), which may limit external generalisability across different manufacturers, detector technologies, and reconstruction algorithms. Accordingly, this study should be interpreted as a single-centre diagnostic accuracy evaluation rather than a universal screening model. Multicentre investigations, such as that conducted by Ni et al. [35], have demonstrated the value of incorporating diverse scanners and acquisition protocols in improving robustness, achieving high internal (DSC ≈ 0.95) and external (DSC ≈ 0.96) performance. Expansion of the dataset across institutions and devices therefore represents an important direction for future research.

Beyond scanner-related constraints, analysis of model errors provides further insight into current limitations. The two false-positive detections observed on the independent test set reflected limitations of the segmentation model rather than the post-processing strategy. In both cases, non-neural anatomical structures adjacent to the mandibular canal—such as dense trabecular bone or the lamina dura surrounding a developing tooth root—were incorrectly segmented, with spurious voxel-level continuity to the main canal. As these structures were predicted as originating from the canal, they were retained during connected-component–based post-processing, which removes only topologically disconnected components. Visual inspection of model predictions before and after post-processing suggested that this step reduced isolated false-positive detections while preserving anatomically plausible bifid branches. These findings highlight the intrinsic difficulty of differentiating neural from non-neural corticated structures on CBCT in anatomically complex regions and suggest that further refinement of anatomical constraints may reduce false-positive detections.

More broadly, challenges inherent to deep learning remain relevant, including overfitting, class imbalance, and limited model interpretability [36]. These issues were addressed through patch-based training, class-weighted loss functions, and expert-guided preprocessing. To mitigate the “black box” concern, the model was trained using clinically meaningful, expert-defined labels, thereby aligning network predictions with anatomical reasoning. While full explainability remains an ongoing challenge, the present design represents a step towards clinically interpretable AI-assisted imaging. Accordingly, this study was designed as a diagnostic accuracy evaluation rather than a comparative or superiority analysis; formal statistical significance testing between models or against human readers was therefore not performed.

Future work should prioritise increased dataset diversity through multicentre collaboration and extend model capabilities beyond detection to classification of BMC subtypes according to the system proposed by Naitoh et al. [11]. Incorporation of advanced architectures, such as attention-gated U-Nets or transformer-based models, may further enhance contextual understanding, while validation in paediatric and orthodontic populations would broaden clinical applicability. Collectively, these developments will be essential for transitioning AI-based MC segmentation from research settings into routine clinical practice.

## Conclusions

This study demonstrates that a multiclass 3D U-Net framework can accurately segment the mandibular canal and detect bifid canal variants on CBCT within a single-institution setting. High Dice scores were achieved for standard canals, with complete detection of bifid variants on the independent test set. These findings support the potential role of automated segmentation as a diagnostic support tool in oral and maxillofacial radiology, particularly for preoperative planning and reduction of iatrogenic nerve injury risk. Further validation across multiple centres and imaging systems is required to establish broader clinical applicability.

## Data Availability

The CBCT imaging data supporting the findings of this study are not publicly available due to institutional data governance and patient confidentiality regulations but may be made available from the corresponding author upon reasonable request.The deep learning pipeline and trained models developed for this study form part of an ongoing research programme and are therefore not publicly available.

## Abbreviations

3D U-Net: Three-dimensional U-Net
AI: Artificial intelligence
AUC: Area under the curve
BMC: Bifid mandibular canal
CBCT: Cone-beam computed tomography
CI: Confidence interval
CT: Computed tomography
DL: Deep learning
DSC: Dice similarity coefficient
FN: False negatives
FP: False positives
IoU: Intersection over Union
Leaky ReLU: Leaky Rectified Linear Unit
MC: Mandibular canal
MONAI: Medical Open Network for AI
MPR: Multi-planar reconstruction
NPV: Negative predictive value
PPV: Positive predictive value
ReLU: Rectified Linear Unit
ROC: Receiver operating characteristic
SD: Standard deviation
TMC: Trifid mandibular canal
TN: True negatives
TP: True positives
